# Post-Traumatic Epilepsy after Sports-Related Concussion: A Case Report

**DOI:** 10.1089/neur.2020.0013

**Published:** 2020-07-23

**Authors:** Justin E. Hellwinkel, Forrest L. Anderson, David P. Trofa, Michael P. Deitrich, Jalen D. Dansby, James M. Noble, Thomas S. Bottiglieri

**Affiliations:** ^1^ Department of Orthopedics, Columbia University Medical Center, New York, New York, USA.; ^2^ Susquehanna University, Selinsgrove, Pennsylvania, USA.; ^3^ Vagelos College of Physicians and Surgeons, Taub Institute for Research on Alzheimer Disease and the Aging Brain, and the G.H. Sergievsky Center, Columbia University, New York, New York, USA.; ^4^ Department of Neurology, Taub Institute for Research on Alzheimer Disease and the Aging Brain, and the G.H. Sergievsky Center, Columbia University, New York, New York, USA.

**Keywords:** brain injury, concussion, post-traumatic epilepsy, sports-related concussion

## Abstract

Sports-related concussions (SRCs) are a public health concern across the United States, and they lead to neurological sequelae that can last long after the event itself. Concussive convulsions at the time of injury are common and rarely require additional workup or treatment. Post-traumatic epilepsy (PTE), however, is a rare phenomenon that can develop after traumatic brain injury and must be managed with adequate medical therapy. Herein we present the case of a 15-year-old football player who developed PTE after an SRC. This condition must be identified through proper education of sports clinicians and those involved in care and management of athletes.

## Introduction

Each year approximately 8 million high-school athletes participate in organized sports in the United States.^1^ Competitive and organized sports are associated with myriad physical, mental, and social benefits to the athlete, but participation also confers risk of injury. Contact and collision sports in particular place athletes at higher risk for neurological injury, including concussion. The rate of sports-related concussions (SRCs) across all high-school sports is estimated to be 4.17 per 10,000 athlete exposures,^2^ with football repeatedly measured as the sport with the highest risk.^3^ The majority of SRCs are uncomplicated and typically resolve within 1–2 weeks.^4^ However, some SRCs are associated with concomitant neurological sequelae, including loss of consciousness and less commonly, post-concussive convulsions.^5^

Post-traumatic epilepsy (PTE), particularly late seizures following traumatic brain injury (TBI),^6,7^ has been well described and accounts for up to 20% of epilepsy in the general population.^8^ It is not clear whether post-traumatic seizures at the time of injury or during the acute/immediate (within 24 h) or early (within the first 7 days) post-concussive period affect the development of PTE.^9,10^ Little is known about the epidemiology or risk factors of PTE in collision sports or in the context of SRC. The rarity of SRC-induced PTE thus represents a diagnostic and therapeutic challenge. Here we describe the case of a 15-year-old male who sustained a football-related concussion and subsequently developed PTE.

## Case Report

A 15-year-old male with a history of multiple, unreported football-related headaches, described feeling “out of it” at the beginning of a high school football game during his junior year. Through the first half of the game, he sustained multiple collisions with other players and an episode of head-to-ground contact. At halftime, the athlete struggled to recall routine stretching exercises and reported difficulty with concentration, dizziness, disorientation, and nausea. During his initial sideline assessment, approximately 25 min after the last contact play, he lost consciousness and sustained a 3- to 4-min generalized tonic-clonic seizure with left hemiparesis in the initial post-ictal period. He spontaneously regained consciousness and was transported to the nearest emergency department, where non-contrast computerized tomography (CT) of the head and neck were unremarkable for acute fracture, intracranial hemorrhage, or edema. He was diagnosed with a concussion and instructed to follow up as an outpatient.

Two days after the event, the patient completed an initial symptom severity inventory and underwent a complete history and physical exam. His symptoms included mild to moderate headaches, dizziness, nausea, fatigue, visual problems, noise sensitivity, light sensitivity, difficulty concentrating, irritability, difficulty remembering, drowsiness, trouble falling asleep, and sleeping more than usual. Past medical history was unremarkable with the exception of multiple, unreported football-related head injuries that resulted in symptoms consistent with SRC, although he was never formally evaluated for these injuries. His physical exam was notable for a positive Spurling test on the right, pain-limiting range of motion in the cervical spine, and left occipital tenderness. The patient did not display any objective signs of dysmetria, weakness, or cranial nerve deficits. He was diagnosed with a cervical spine sprain, concussion with loss of consciousness, and post-concussive seizure. Treatment included academic modifications, a low-intensity heart rate-controlled home exercise program, proper sleep hygiene, and avoidance of contact sports. At follow-up 2 weeks later, the patient's symptom severity decreased compared with his initial survey results and he continued with the same treatment regimen.

The patient had persistent post-concussion symptoms and had a witnessed tonic-clonic seizure after neuropsychological testing 10 weeks after injury. He experienced two more unprovoked tonic-clonic seizures in the first 4 months post-injury. Magnetic resonance imaging (MRI) of the brain without contrast did not reveal focal abnormality and electroencephalography (EEG) demonstrated right greater than left temporal slowing and right temporal, sharp waves. He began oxcarbazepine therapy and was followed closely by a sports physician, neurologist, neuropsychologist, cognitive behavioral therapist, physical therapist, athletic trainer, and neuroopthalmologist. His symptoms slowly improved over time and he had one seizure event since starting antiepileptic medication. One year after the event, he continues to experience mild, but improved post-concussive symptoms.

## Discussion

PTE has been described following focal brain trauma.^11^ To date, only nine cases of the development of PTE due to SRC have been described,^12^ although the prevalence of this condition is likely greater due to underreporting of concussive symptoms in athletes.^13^ Diagnosing PTE can be difficult because of the need to establish a temporal association with head trauma. Differentiation between post-concussive convulsions and PTE is necessary to guide treatment and can be distinguished primarily by the timing of seizure events. Post-concussive convulsions are thought to occur in approximately 1 of 70 concussions and occur immediately following concussive brain injury.^14^ Although the neurophysiological basis for convulsions in this setting is uncertain, evidence supports transient release of excitatory neurotransmitters, followed by global cortical inhibition, resulting in seizure-like activity.^15^ However, immediate or early seizures poorly predict future seizure activity and imaging studies are typically unrevealing for underlying pathology. When treating the concussions of these individuals, no additional antiepileptic therapy is recommended. One recent comprehensive review reported on 130 athletes with concussive convulsions (at the time of SRC) over an average follow-up period of 3.3 years.^14^ Among these athletes, only nine (6.9%) had new-onset epilepsy following the event, suggesting a low incidence of PTE following immediate post-concussion seizures.

Elements of this case highlight typical but also unique challenges in the management of contact sports athletes. Minimized SRC symptoms, such as “football headaches” reported by this patient, highlight the continued problem and importance of timely and open symptom reporting. A combination of better objective tools for concussion surveillance and continued education efforts for players, parents, coaches, and officials could mitigate these risks.

Relatedly, establishing a diagnosis of PTE is challenging given unreported TBI symptoms in athletes. Epilepsy in athletes could follow exposure to brain injury in some contact and collision sports and without a known diagnosis of SRC or other TBI. Moreover, it is not yet established if athletes who sustain SRC with associated convulsions are at higher risk for developing PTE than those who do not experience immediate convulsions. The development of PTE, and less often focal TBI, has been recognized in boxers, a sports population with frequent SRC; boxers with PTE often demonstrate cerebral atrophy on CT imaging and epileptogenic EEG.^12^

Recommendations for the workup of PTE in SRC, aside from close longitudinal clinical monitoring, are patient-specific but may include advanced brain imaging or EEG if there is concern for focal neurological deficit or epileptiform activity. Although in this case there was no radiographic evidence of focal injury on MRI, mild TBI often lacks abnormal imaging signal. An important unresolved issue raised by this case is the possibility that focal injury and not just diffuse injury is within the spectrum of second impact syndrome.^16^ Within the broader spectrum of TBI and PTE, treatment is centered around antiepileptic medication and neurorehabilitation.^17^ Although the natural history of PTE in SRC is uncertain, treatment-refractory PTE following the broader TBI spectrum occurs in up to 53% of patients.^8^

## Conclusion

PTE may require specific attention in youth athletes given its potential impact on critically important and ongoing social, cognitive, and academic development timelines. School counselors and health workers, athletic trainers, neuropsychologists, neurologists, and concussion specialists are concussion care team members treating athletes who may be in a position to support and monitor athletes with convulsions in the post-concussive period. The epidemiology of post-SRC PTE is not well established but could be enabled by ongoing large-scale efforts in collegiate SRC research, as well as study of long-term outcomes of other forms of diffuse mild TBI, particularly in youth and emerging adults. Management of SRC-induced PTE will evolve as the pathophysiology of PTE is better understood.

Permission for this case report was granted by the patient and family members.

## Abbreviations Used

CT: computerized tomography
EEG: electroencephalography
MRI: magnetic resonance imaging
PTE: post-traumatic epilepsy
SRC: sports-related concussion
TBI: traumatic brain injury

## References

[1] National Federation of State High School Associations. (2019). 2018-19 High School Athletics Participation Survey. https://www.nfhs.org/sports-resource-content/high-school-participation-survey-archive/ (Last accessed 719, 2020)

[2] Kerr, Z.Y., Chandran, A., Nedimyer, A.K., Arakkal, A., Pierpoint, L.A., and Zuckerman, S.L. (2019). Concussion incidence and trends in 20 high school sports. Pediatrics 144, e20192180 31615955 10.1542/peds.2019-2180

[3] Noble, J.M., and Hesdorffer, D.C. (2013). Sport-related concussions: a review of epidemiology, challenges in diagnosis, and potential risk factors. Neuropsychol. Rev.23, 273–284 10.1007/s11065-013-9239-0 24242889

[4] McCrory, P., Meeuwisse, W., Dvorak, J., Aubry, M., Bailes, J., Broglio, S., Cantu, R.C., Cassidy, D., Echemendia, R.J., Castellani, R.J., Davis, G.A., Ellenbogen, R., Emery, C., Engebretsen, L., Feddermann-Demont, N., Giza, C.C., Guskiewicz, K.M., Herring, S., Iverson, G.L., Johnston, K.M., Kissick, J., Kutcher, J., Leddy, J.J., Maddocks, D., Makdissi, M., Manley, G.T., McCrea, M., Meehan, W.P., Nagahiro, S., Patricios, J., Putukian, M., Schneider, K.J., Sills, A., Tator, C.H., Turner, M., and Vos, P.E. (2017). Consensus statement on concussion in sport: the 5(th) International Conference on Concussion in Sport held in Berlin, October 2016. Br. J. Sports Med. 51, 838–847 28446457 10.1136/bjsports-2017-097699

[5] Davis-Hayes, C., Baker, D.R., Bottiglieri, T.S., Levine, W.N., Desai, N., Gossett, J.D., and Noble, J.M. (2018). Medical retirement from sport after concussions: a practical guide for a difficult discussion. Neurol. Clin. Pract. 8, 40–47 29517059 10.1212/CPJ.0000000000000424 PMC5839677

[6] Ding, K., Gupta, P.K., and Diaz-Arrastia, R. (2016). Epilepsy after traumatic brain injury. in: *Translational Research in Traumatic Brain Injury*. G. Laskowitz, and G. Grant (eds). Boca Raton, FL: CRC Press/Taylor and Francis Group 26583175

[7] Fisher, R.S., Acevedo, C., Arzimanoglou, A., Bogacz, A., Cross, J.H., Elger, C.E., Engel, J. Jr., Forsgren, L., French, J.A., Glynn, M., Hesdorffer, D.C., Lee, B.I., Mathern, G.W., Moshe, S.L., Perucca, E., Scheffer, I.E., Tomson, T., Watanabe, M., and Wiebe, S. (2014). ILAE official report: a practical clinical definition of epilepsy. Epilepsia 55, 475–482 24730690 10.1111/epi.12550

[8] Lucke-Wold, B.P., Nguyen, L., Turner, R.C., Logsdon, A.F., Chen, Y.W., Smith, K.E., Huber, J.D., Matsumoto, R., Rosen, C.L., Tucker, E.S., and Richter, E. (2015). Traumatic brain injury and epilepsy: underlying mechanisms leading to seizure. Seizure 33, 13–23 26519659 10.1016/j.seizure.2015.10.002 PMC12767287

[9] Mendonca, G.S., and Sander, J.W. (2019). Post traumatic epilepsy: there is still much to learn. Arq. Neuropsiquiatr. 77, 373–374 31314837 10.1590/0004-282X20190068

[10] Qian, C., Lopponen, P., Tetri, S., Huhtakangas, J., Juvela, S., Turtiainen, H.M., Bode, M.K., and Hillbom, M. (2014). Immediate, early and late seizures after primary intracerebral hemorrhage. Epilepsy Res. 108, 732–739 24661429 10.1016/j.eplepsyres.2014.02.020

[11] Frey, L.C. (2003). Epidemiology of posttraumatic epilepsy: a critical review. Epilepsia 44, 11–17 10.1046/j.1528-1157.44.s10.4.x 14511389

[12] Maximov, G.K., and Moximov, Konatantin G. (2008). Repeated head trauma—decisive factor of delayed onset epilepsy in boxers. Pharmacologyonline 2, 18–21

[13] Wallace, J., Covassin, T., Nogle, S., Gould, D., and Kovan, J. (2017). Knowledge of concussion and reporting behaviors in high school athletes with or without access to an athletic trainer. J. Athl. Train. 52, 228–235 28387561 10.4085/1062-6050-52.1.07 PMC5384820

[14] Kuhl, N.O., Yengo-Kahn, A.M., Burnette, H., Solomon, G.S., and Zuckerman, S.L. (2018). Sport-related concussive convulsions: a systematic review. Phys. Sports Med. 46, 1–7 10.1080/00913847.2018.1419775 29280684

[15] Ban, V.S., Botros, J.A., Madden, C.J., and Batjer, H.H. (2016). Neurosurgical emergencies in sports neurology. Curr. Pain Headache Rep. 20, 55 27534950 10.1007/s11916-016-0586-4 PMC4988993

[16] Stovitz, S.D., Weseman, J.D., Hooks, M.C., Schmidt, R.J., Koffel, J.B., and Patricios, J.S. (2017). What definition is used to describe second impact syndrome in sports? A systematic and critical review. Curr. Sports Med. Rep. 16, 50–55 10.1249/JSR.0000000000000326 28067742

[17] Conder, A., Conder, R., and Friesen, C. (2020). Neurorehabilitation of persistent sport-related post-concussion syndrome. NeuroRehabilitation 46, 167–180 32083597 10.3233/NRE-192966

